# High-Precision Colorimetric Sensing by Dynamic Tracking of Solvent Diffusion in Hollow-Sphere Photonic Crystals

**DOI:** 10.34133/2022/9813537

**Published:** 2022-05-06

**Authors:** Qianqian Fu, Jianping Ge, Chen Chen, Zichen Wang, Fan Yang, Yadong Yin

**Affiliations:** ^1^Department of Chemistry, University of California, Riverside, California, USA CA 92521; ^2^School of Chemistry and Molecular Engineering, Shanghai Key Laboratory of Green Chemistry and Chemical Processes, East China Normal University, Shanghai, China 200062; ^3^Institute of Eco-Chongming, Shanghai, China 202162

## Abstract

Expensive instruments and complicated data processing are often required to discriminate solvents with similar structures and properties. Colorimetric sensors with high selectivity, low cost, and good portability are highly desirable to simplify such detection tasks. Herein, we report the fabrication of a photonic crystal sensor based on the self-assembled resorcinol formaldehyde (RF) hollow spheres to realize colorimetric sensing of polar solvents, including homologs and isomers based on the saturated diffusion time. The diffusion of solvent molecules through the photonic crystal film exhibits a unique three-step diffusion profile accompanied by a dynamic color change, as determined by the physicochemical properties of the solvent molecules and their interactions with the polymer shells, making it possible to accurately identify the solvent type based on the dynamic reflection spectra or visual perception. With its superior selectivity and sensitivity, this single-component colorimetric sensor represents a straightforward tool for convenient solvent detection and identification.

## 1. Introduction

Monitoring chemicals and organic pollutants has become very important in areas such as the food industry, agriculture, and environment protection. There is an increasing demand for developing novel sensors with high sensitivity, low cost, and good portability. Materials based on photonic crystals (PCs) are highly attractive for this purpose because their optical properties are determined by their nanoscale periodic arrangement and dielectric environment, which change in response to many physical and chemical external stimuli. In addition to sensing [1–8], they are also useful for a broad range of applications, including printing [9–13], anticounterfeiting [14–16], display units [17–20], photocatalysis [21–23], and solar energy harvesting [24, 25]. In particular, they have been extensively explored as active components in sensors by offering simple optical and visual means to determine physicochemical parameters of various analytes [26, 27].

Generally, photonic sensors detect the optical signals associated with the changes in refractive index and lattice constant of PCs upon their exposure to solvents and gases [28, 29]. They often feature an inverse opal structure, or they are fabricated using hollow spheres or porous materials because a large volume fraction of cavities can greatly increase the effective refractive index, thereby improving sensitivity [30–33]. An additional benefit of using porous structures is that it can increase the specific surface area, which improves the analyte adsorption and allows the sensor to distinguish between molecules of close similarities, such as isomers [31]. Dynamic reflection spectra (DRS) have recently been proposed to distinguish solvents with similar structures during their diffusion through photonic materials [34–36]. Swellable materials with specific interactions with analytes are used to drive distinct optical responses determined by the swelling behavior, refractive index change, and diffusion rate [37, 38]. However, this method still requires a spectrometer and sophisticated data processing, and considerable efforts are required to read out the signals from the DRS patterns. Practical applications highly prefer colorimetric sensors that can discriminate solvents with similar structures by visual inspection or digital-camera color imaging system.

In this work, we report the development of a photonic crystal sensor based on self-assembled resorcinol-formaldehyde (RF) hollow spheres for visual detection of solvents according to their saturated diffusion time. The RF hollow spheres hold the key to successful solvent detection by enabling a unique three-step diffusion profile, including solvent infiltration to the interparticle voids, swelling of the polymer shell, and filling of the cavity inside the RF shells. Both *in situ* monitoring and theoretical simulation suggest that shell swelling is the rate-determining step. The distinct changes in the structural color associated with the diffusion steps make it convenient to track the molecular diffusion process and visually identify the saturated diffusion time. The saturated diffusion time is determined not only by the physicochemical property of the solvent molecules but also by their interaction with the RF shell, making this photonic sensor highly selective and sensitive. The promise of these photonic sensors in practical applications has been demonstrated by qualitative identification of polar solvents and quantitative determination of water and methanol concentration in alcoholic systems.

## 2. Results and Discussion

**Figure 1:**
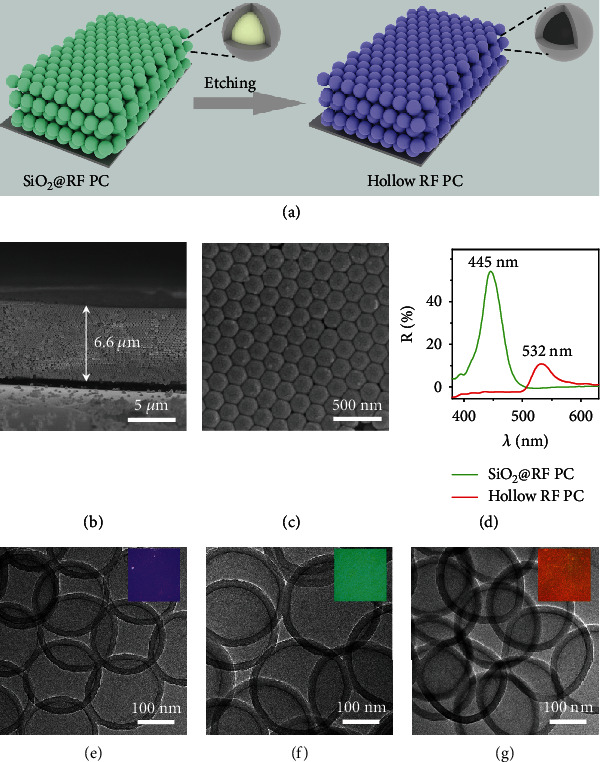
Fabrication of hollow RF sphere photonic crystals. (a) Scheme illustrating the fabrication process of photonic crystals consisting of hollow RF spheres. (b, c) SEM images of PC film of hollow RF spheres: cross-section (b) and top-view (c). (d) Reflectance spectra of PC films before and after removing silica. (e–g) TEM images of hollow RF spheres with diameters of 210 nm (e), 245 nm (f), and 275 nm (g). The insets are dark-field optical microscopy images of the corresponding PCs.

The photonic sensors were prepared by etching silica from the preassembled SiO_2_@RF photonic crystals (Figure 1(a), S1). Monodispersed SiO_2_ spheres were first synthesized through a sol-gel process and then coated with an RF shell [39]. The resulting SiO_2_@RF core-shell spheres were assembled into liquid PCs through a supersaturation-induced precipitation process [40]. Typically, a highly concentrated suspension of monodispersed SiO_2_@RF particles (30 vol%) in ethylene glycol (EG) was spread on a PET film pretreated with O_2_ plasma. The liquid film was allowed to sit without disturbance at room temperature for 10 minutes to form metastable colloidal microcrystals. After drying at 90°C for 30 minutes, it became a solid film showing green structural color. Finally, removing the silica cores by HF etching produced a PC film composed of highly ordered hollow RF spheres (Figures 1(b) and 1(c)), accompanied by a blue shift of reflection peak from 532 nm to 445 nm (Figure 1(d)). The resultant film displayed a uniform purple structural color. The hollow RF spheres of a typical sample were further characterized by TEM imaging, showing a diameter of 185 nm and shell thickness of 15 nm. The size and shell thickness of hollow spheres can be readily tuned by the sizes of SiO_2_ cores and the concentration of RF precursors used during the coating process. Here, we synthesized hollow spheres with different diameters (210, 245, and 275 nm) while maintaining the thickness of the RF shells at ~15 nm (Figures 1(e)–1(g), Figure S2). Accordingly, assembling such hollow spheres produced purple, green, and orange PC films, as shown in the insets.

**Figure 2:**
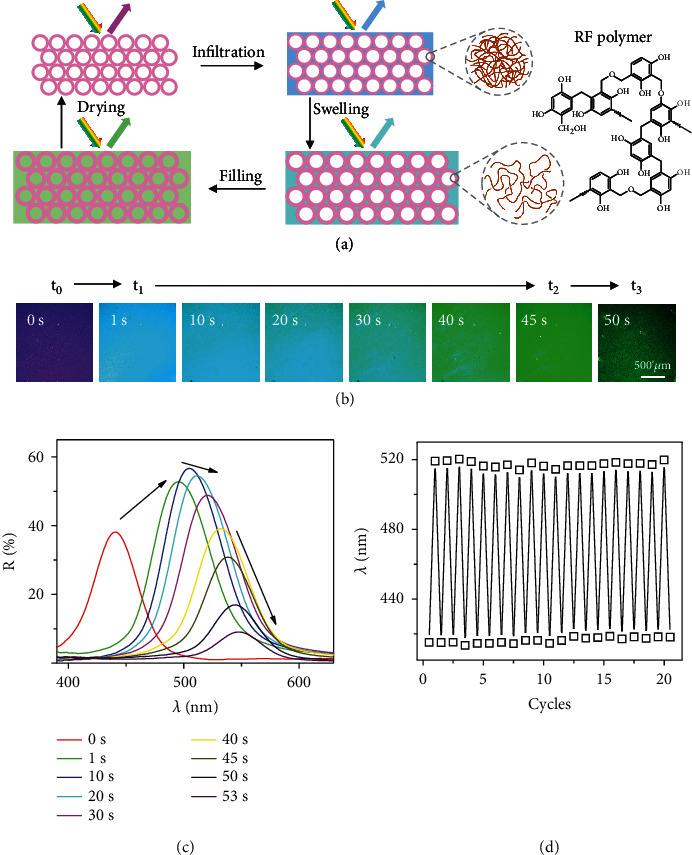
Typical three-step diffusion process of solvents. (a) Scheme illustrating the three-step diffusion of solvents in the hollow-sphere PCs. (b, c) Dark-field optical microscopy images (b) and corresponding reflectance spectra (c) during ethanol diffusion in the PC film. (d) Changes of reflection wavelength in response to repeated infiltration with ethanol and then drying for 20 cycles.

The PC film exhibits an interesting three-step color change upon contact with solvents (Figure 2(a), Video S1). In the first step, the application of ethanol to the PC film led to an immediate color change from purple to bright blue (within a second), as observed in the dark-field optical microscope (Figure 2(b)). It is interpreted as the quick solvent infiltration to the interstices of the hollow spheres, increasing the average refractive index and redshifting the reflection (Figure 2(c)). In the second step, the PC film gradually changed from blue to bright green in the following several tens of seconds, attributed to the swelling of the RF shells by the solvent and the accompanied size increase. In the third step, the PC film turned dark green within a few seconds with a significant drop in brightness, indicating that the solvent diffused through the outer shell and filled the interior of the hollow spheres. In addition, the coordinates in the Lab color space of the ethanol diffusion process were continually measured to provide direct chroma information. The lightness (*L*∗) of the PC film also exhibited three stages, and a sharp decrease of lightness in the last filling step was observed, making it convenient for tracking diffusion by naked eyes (Figure S3). Moreover, the film could recover to its original purple after drying and still showed a good solvent response after 20 wetting and drying cycles, proving the high reversibility of the photonic response (Figure 2(d)).

**Figure 3:**
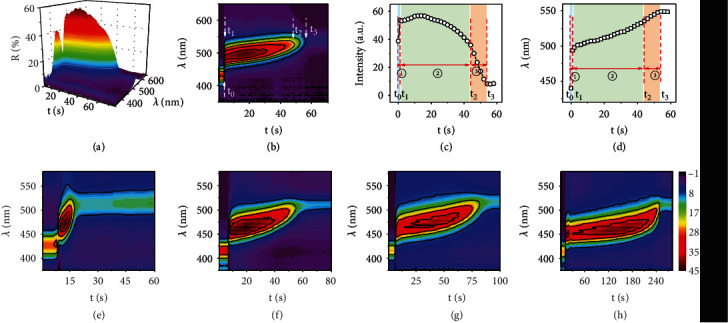
Dynamic tracking of solvents diffusion by DRS. (a, b) The dynamic reflection spectrum of solvent diffusion process in the form of a 3D surface map (a) and a contour map (b). (c, d) The intensity (c) and wavelength (d) signals extracted from the contour map. (e–h) DRS patterns resulting from solvent diffusion in PC films that were aged for 1 min (e), 5 min (f), 10 min (g), and 60 min (h) at 70°C.

The three-step diffusion process was further confirmed by measuring DRS. As shown in Figures 3(a) and 3(b), in a typical response to ethanol, a series of reflection spectra were continuously recorded upon the addition of ethanol to the film, forming a 3D surface map with time (*t*), reflection wavelength (*λ*), and the intensity (*R*) as *x*-, *y*-, and *z*-axis, respectively. It can be further plotted as a surface contour map by converting the reflection intensity into color signals. According to the change of reflection intensity and shift of reflection wavelength during the diffusion process, we could clearly identify the three steps of solvent diffusion, consistent with the direct observations in the optical microscope (Figures 3(c) and 3(d)). In the infiltration step (*t*_0_⟶*t*_1_), the reflection redshifted from 440 nm to 494 nm within one second, accompanied by a significant enhancement of the reflection intensity, caused by the increase of effective refractive index and the increase of refractive index contrast between the particle interstices and the cavities within shells. In the swelling step (*t*_1_⟶*t*_2_), the reflection slightly redshifted in 45 seconds and maintained a relatively high intensity, which came from the swelling of the polymer shells of hollow spheres. In the final step (*t*_2_⟶*t*_3_), the reflection intensity dropped sharply from 36% to 8% and finally reached an equilibrium value, corresponding to the solvent's penetration and filling of the hollow spheres. As a result, the decrease of RI contrast leads to a significant decrease in the brightness of the structural color. Finally, the time for saturated solvent diffusion (*t*_3_) could be determined from the DRS patterns, also by visual inspection due to the significant change in the structural color and brightness in the last step.

The swelling of the RF shell was identified as the rate-determining step in the entire solvent diffusion process, as confirmed by comparing the diffusion profiles and the corresponding DRS patterns when the solvents wet the films made from RF shells of different crosslinking degrees by aging at 70°C for varying periods [41]. As shown in Figures 3(e)–3(h) and Figure S4, when the heating time increased from 1 min to 60 min, the time for swelling (*t*_2_‐*t*_1_) increased from 4 s to 220 s, and the saturated diffusion time (*t*_3_) extended from 12 s to 248 s, consistent with the increased crosslinking of the polymer shells. However, both the first infiltration step and the last filling step were relatively quick with little time difference for different films. The overall solvent diffusion was dominated by the swelling step, which is, therefore, the rate-determining process. In addition, PC films assembled from different RF shell thicknesses (17.5 nm, 23 nm, and 29 nm) were utilized to illustrate the rate-determining step. As shown in Figure S5, the saturated diffusion time of ethanol in PC films increased from 61 s to 96 s as the RF shell thickness increased from 17.5 nm to 29 nm. The increase in diffusion time was mainly caused by extending the second swelling step, further confirming that the swelling step is the rate-determining step.

**Figure 4:**
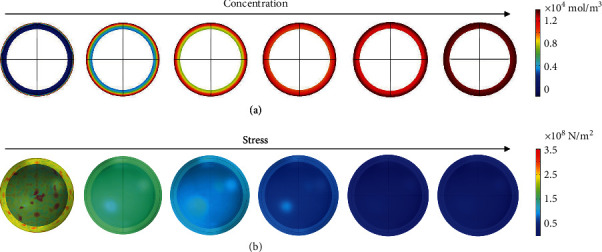
Simulation of DMSO diffusion process. Simulation of (a) solvent concentration and (b) shell deformation and stress change during the diffusion of DMSO in an RF shell.

To gain insight into the swelling process, we conducted a finite element simulation to study the solvent diffusion behavior through the RF polymer shells. Here, we used a combination of diffusion and polymer elastic models to describe the diffusion of a marker solvent, DMSO, in hollow spheres. The simulation is based on two assumptions: (i) the swelling of the polymer shell takes place along with the solvent diffusion, and (ii) the swelling and diffusion terminate as the inner and outer RF shells reach equal and saturated solvent concentration, and then, the solvent penetrates the shell into the cavity of the hollow sphere. As shown in Figure 4(a), the solvent gradually diffuses from the outmost layer to the inner layer until it is distributed in the entire RF shell and reaches the saturated concentration. At the same time, the RF shell shows an apparent swelling behavior with the shell expanding until a new equilibrium state is reached due to the decreasing integral stress of the sphere (Figure 4(b)). The simulated particle diameter increases with the swelling of the RF shell, leading to a shift in the calculated reflection wavelength. Consistent with the experimental results (Figure S6), the simulation suggests that the diffusion of solvent molecules in the PC film can be precisely tracked by recording the DRS patterns or visual inspection. Since the swelling is the rate-determining step and is tightly related to the properties of polymer network and solvents, the saturated diffusion time (*t*_3_) obtained from the optical tracking of a specific solvent diffusion in the PC film can be used as the primary parameter to characterize the solvent molecules.

**Figure 5:**
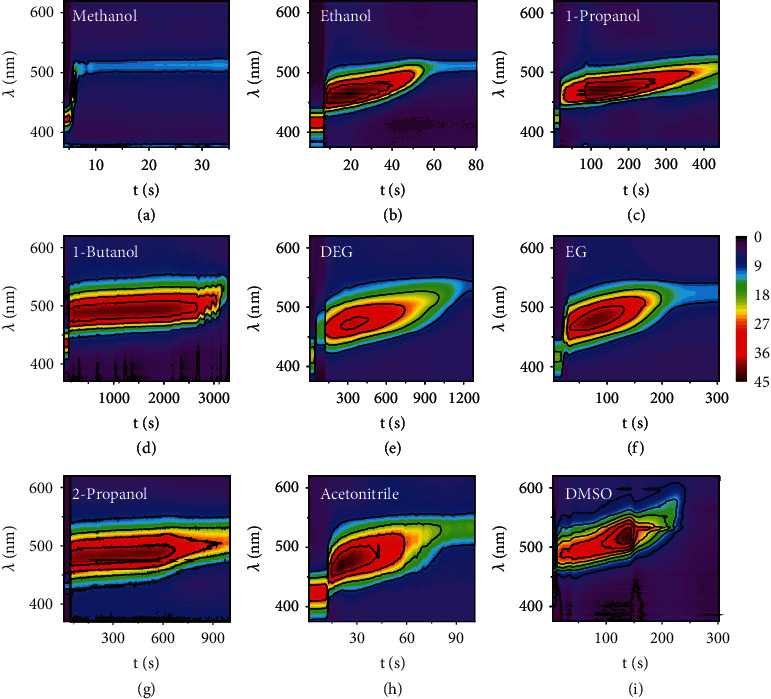
DRS patterns of various polar solvents. DRS patterns resulting from the diffusion of various solvents in PC films: (a) methanol, (b) ethanol, (c) 1-propanol, (d) 1-butanol, (e) DEG, (f) EG, (g) 2-propanol, (h) acetonitrile, and (i) DMSO.

The ability to track the dynamic diffusion process enables the further development of a photonic solvent sensor that can distinguish polar solvents based on the saturated diffusion time. We investigated several solvent properties, such as dielectric constant, molecular size, and viscosity on the saturated diffusion time (Figure 5). First, the diffusion behaviors of alcohol homologs were investigated, exhibiting distinct DRS patterns as shown in Figures 5(a)–5(d). The entire diffusion of methanol, ethanol, 1-propanol, and 1-butanol took 1 s, 50 s, 421 s, and 3600 s, suggesting that the alcohols with smaller sizes and larger dielectric constants may diffuse faster in the PC films. In addition, viscosity greatly influences solvent diffusion. For example, the diffusion time of DEG (1192 s) is much larger than that of methanol because of its higher viscosity (35.7 mPa·s) than the latter (0.595 mPa·s), although their dielectric constants are close (31.7 for DEG vs. 31.2 for methanol). Similarly, the slower diffusion of EG than acetonitrile can also be attributed to EG's higher viscosity. Therefore, the significant difference in the diffusion time of different solvents makes their colorimetric discrimination possible.

**Figure 6:**
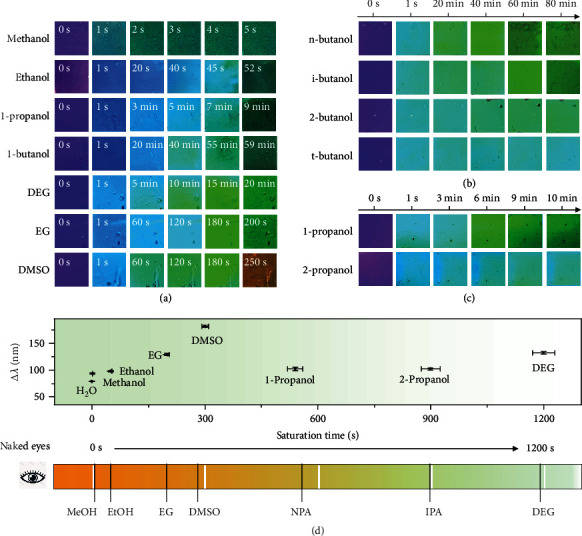
Colorimetric sensing of various solvents. (a) Digital photos of PC films infiltrated by methanol, ethanol, 1-propanol, 1-butanol, DEG, EG, and DMSO. (b, c) Digital photos of PC films infiltrated by butanol isomers (b) and propanol isomers (c). (d) Plot showing the wavelength shift vs. the saturated diffusion time for different solvents (upper panel, obtained from DRS) and the saturated diffusion time of solvents observed by naked eyes (bottom panel).

While the DRS patterns could provide rich information for discriminating solvents, visual sensing is more favorable in practical applications, which is possible by taking a series of digital photos instead of spectra to record the diffusion process of polar solvents in PC films. As shown in Figure 6(a), the PC films immediately turned from purple to bright blue in all cases. In the final stage of diffusion, the PC films' structural color brightness and saturation decreased significantly, turning to dark green or dark red in a relatively short period. In contrast to other solvents, the PC film exposed to DMSO appeared dark orange at the final state due to DMSO's higher refractive index and stronger swelling ability to the RF shell. Thanks to the color and brightness change of the photonic films, the saturation time of solvent diffusion could be readily determined by naked eyes (Video S3). The saturation time of methanol, ethanol, 1-propanol, and 1-butanol was 1 s, 52 s, 7 min, and 59 min, respectively, close to the results obtained from the DRS patterns. More alcohol homologues from C5 to C8 (1-pentanol, 1-hexanol, 1-heptanol, and 1-octanol) could also be distinguished by the naked eyes. Here, the analytes were mixed with methanol in the proportion of 1 : 1 to speed up the measurement process. As shown in Figure S7, a longer carbon chain corresponds to a longer saturated diffusion time. It is worth noting that the visual detection of ethanol homologs is difficult to achieve in traditional PCs composed of solid particles. In addition, the saturated diffusion times of other polar solvents such as EG, DEG, and DMSO can also be obtained using this method, consistent well with previous results, further verifying the accuracy and feasibility of the visual solvent sensing. The unique combination of dynamic color response, reversibility, high selectivity, and easy reading makes the photonic solvent sensor suitable for practical applications.

The high selectivity of the novel PC films strongly relies on the difference in the diffusion coefficient of the solvents. According to the Vrentas–Duda free-volume theory, the diffusion of solvents in polymer could be described by solvent self-diffusion coefficient (*D*_1_) and mutual diffusion coefficient (*D*). The latter can be expressed by equation (1), where *φ*_*s*_ is the volume fraction of solvent and *χ* is the Flory-Huggins interaction parameter. Characterizing the interaction of the polymer and solvent molecules, *χ* can be calculated by equation (2), where *V*_*s*_ is the molar volume of the solvent molecule and *δ*_*s*_ and *δ*_*p*_ are the solubility parameters of solvent and polymer, respectively [42–44].

(1) $$\begin{array}{c} D=D_{1}{\left(1-\varphi_{s}\right)}^{2}\left(1-2\chi \varphi_{s}\right), \end{array}$$

(2) $$\begin{array}{c} \chi =\frac{V_{s}}{RT}{\left(\delta_{s}-\delta_{p}\right)}^{2}+0.35. \end{array}$$

Generally, the self-diffusion coefficient is related to the solvent molecules' viscosity, size, rigidity, configuration, and other parameters. A solvent with lower viscosity and smaller molecular size has a faster diffusion rate in a polymer. Therefore, water and methanol with small kinetic diameters induce the fastest response. The Flory-Huggins theory also suggests that the interaction between solvent and polymer greatly influences diffusion. When the solvent and polymer have similar solubility parameters, the solvent shows a stronger swelling ability to the polymer [21]. If the volume change due to swelling is neglected, the wavelength shift of the second diffusion step calculated by only considering the refractive index change is always smaller than the experimental results. Such a disagreement demonstrates the solvents' important contribution to the photonic response by swelling the RF shells (Table S2). Since DMSO has a close solubility parameter to RF, it exhibits the strongest swelling ability to RF polymer among all the tested solvents. In summary, the diffusion behavior of solvent in a polymer is determined by the combined effect of several parameters, including the solvent's physicochemical properties and the solvent-polymer interaction. These multidimensional parameters allow for differentiating solvents and endow the photonic sensor with higher selectivity.

We further demonstrate the effectiveness of the photonic sensor in distinguishing isomers with subtle differences in configuration, viscosity, and polarity, first using butanol isomers as examples, including 1-butanol, 2-butanol, iso-butanol, and tert-butanol. It took a relatively long time for butanol isomers to swell the polymer shell due to their relatively long chain and weak interaction with RF polymer. Their saturation time by visual detection increased in the order of n-butanol, iso-butanol, 2-butanol, and tert-butanol. As shown in Figure 6(b), the film with 1-butanol turned dark green after 60 minutes, while it took 80 minutes for iso-butanol to complete the diffusion process. In contrast, the structural color of PC film with tert-butanol remained almost constant after 80 minutes, indicating the slowest diffusion rate of all. As for propanol isomers, the saturation time of 1-propanol and 2-propanol was 530 s and 920 s, respectively. We can see that the PC film with 1-propanol turned dark green while the film with 2-propanol still maintained bright blue after 9 minutes (Figure 6(c)). Moreover, 1,4-butanediol and 1,3-butanediol could also be visually detected based on the significant difference in the diffusion process (Figure S7). The high sensitivity and directly visible optical properties make this method suitable for isomer detection, which is difficult to achieve by traditional sensors. Figure 6(d) summarizes the PC film's wavelength shift and saturation time in response to typical polar solvents, providing criteria for photonic solvent sensing.

**Figure 7:**
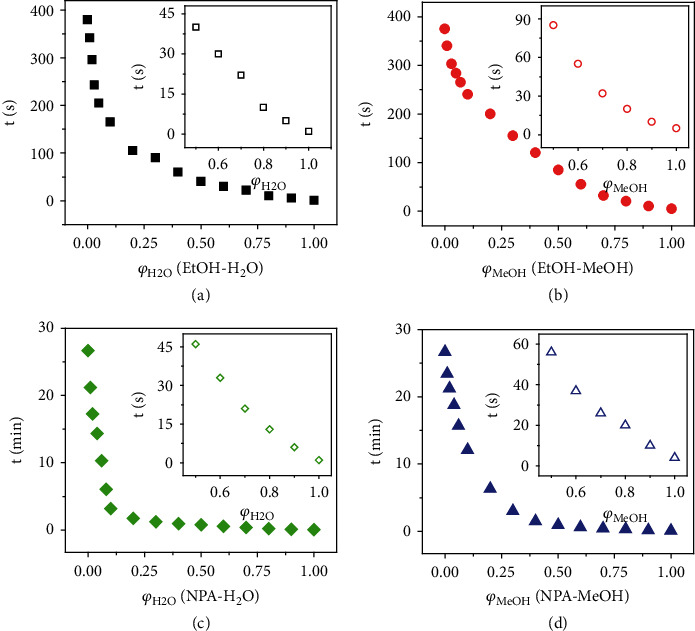
Quantitative determination of component concentration. The saturated diffusion time of various solvent mixtures in PC films: (a) ethanol and water, (b) ethanol and methanol, (c) 1-propanol and water, and (d) 1-propanol and methanol. The insets are the close-ups of the plots for the volume fraction between 0.5 and 1. The PC films were predried for 24 h at 70°C.

In addition to the qualitative identification of polar solvents, the PC films also allow quantitative determination of component concentration in a mixed solvent. As a demonstration, we tested the diffusion behavior of ethanol-water and ethanol-methanol systems in the PC films. For extending the diffusion process, the PC films were pretreated at 70°C for 24 h.  Figure 7(a) shows an exponential decline in saturation time as water content increased from 0% to 10%, which was highly desirable for sensing small amounts of water. When the volume fraction of water was as low as 0.5%, its diffusion time dropped to 342 s from 380 s for pure ethanol. The insets are the close-ups of the volume fraction range of water from 50% to 100%, indicating the possibility of water sensing over the entire range. Similarly, this sensor also allows visual detection of methanol in ethanol-methanol mixtures (Figure 7(b)). In the propanol-water and propanol-methanol mixtures, the saturation time declined more sharply in the low water or methanol regime, indicating high sensing precision and sensitivity (Figures 7(c) and 7(d)). With the flexibility for visual determination and the possibility of extending their applications to other mixtures, the current photonic sensor can be used as test strips, providing a convenient and attractive alternative to the complex traditional water/methanol sensing methods such as Karl-Fischer titration and GC.

## 3. Conclusion

A new type of photonic sensor has been developed using hollow-sphere-based photonic crystals to distinguish solvents with similar properties and structures by determining the saturated diffusion time. The diffusion of solvents in the PC films composing hollow spheres with swellable polymer shells can be divided into three steps: infiltration of solvents to interstices between hollow spheres, swelling of the polymer shells, and filling of the hollow cavities. An advantageous feature worth highlighting is that the diffusion of solvent molecules can be tracked in real time by recording DRS patterns or visual perception to monitor the dynamic structural color changes. The combination of multiple factors, including the physicochemical properties of analytes and the distinct analyte-polymer interactions, determines their unique diffusion profiles, bestowing the photonic sensor with superior selectivity and sensitivity. As a result, solvents with similar structures and properties such as homologs and even isomers can be precisely distinguished by naked-eye observation. Without relying on expensive instruments and complex data analysis, this low-cost, easy-to-read photonic sensor has broad application prospects for environmental monitoring, anticounterfeiting, and quantitative alcohol analysis.

## 4. Materials and Methods

### 4.1. Materials

Tetraethylorthosilicate (TEOS, 98%), aqueous ammonia (28%), dimethyl sulfoxide (DMSO, 99%), acetonitrile (81.0-82.0%), methanol (99.5%), and formaldehyde aqueous solution (37-40%) were purchased from Sinopharm Chemical Reagent Co., Ltd. PVP (Mw = 10000), resorcinol (99%), ethylene glycerol (EG, 99%), diethylene glycerol (DEG, 99%), n-butanol (99.5%), 2-butanol (99%), tert-butanol (99.5%), and iso-butanol (99%) were purchased from Aladdin Co., Ltd. Ethanol (99.9%) and 2-propanol (99.9%) were purchased from J&K Co., Ltd. All chemicals were used directly as received without further purification.

### 4.2. Synthesis of SiO_2_ Colloidal Particles

Firstly, SiO_2_ nanoparticles with a diameter of 185 nm were synthesized by a modified Stöber method. Typically, ethanol (100 mL), H_2_O (7 mL), and NH_3_∙H_2_O (4 mL) were mixed and stirred for 15 minutes. Then, TEOS (8 mL) was quickly injected into the solution and allowed to react for 3 hours. The SiO_2_ particles were collected by centrifugation, washed with ethanol three times, and finally dispersed in 10 ml of H_2_O.

### 4.3. Synthesis of SiO_2_@RF Core-Shell Particles

The surface of SiO_2_ particles was modified with PVP before RF coating. The as-prepared SiO_2_ suspension (1 mL) was dispersed in PVP solution (Mn = 10000, 5 mg/mL) and kept under stirring for 6 h. Then, the PVP modified particles were separated by centrifugation and redispersed in 28 mL of water containing 30 mg of resorcinol, 42 *μ*L of formaldehyde, and 100 *μ*L of diluted ammonia (2.8 wt% in water). The solution was then heated up to 60°C for 2 h and boiled at 100°C for 2 h. The particles were centrifuged and washed with water three times.

### 4.4. Fabrication of Hollow RF Sphere Photonic Crystals

SiO_2_@RF particles (0.03 cm^3^) dispersed in ethanol (1 mL) were firstly mixed with EG (0.07 mL) to form a homogeneous suspension. After being heated at 90°C for 2 h, the suspension was concentrated, and the final volume fraction of SiO_2_@RF was 30%. Then, the liquid precursor was spread on a plastic film, which was treated in advance with plasma. The liquid film was placed under room temperature for 20 minutes without disturbance to form liquid photonic crystals. Subsequently, solid SiO_2_@RF photonic crystal film was obtained by drying the liquid film under 70°C for 30 minutes. The as-prepared film was then immersed in HF solution (2%) for 10 minutes to etch the SiO_2_ cores. The resultant hollow RF sphere photonic crystals exhibited bright purple color.

### 4.5. Characterizations

The dynamic reflection spectra were continuously recorded using an Ocean Optics HR 2000CG-UV-NIR spectrometer. A standard reference aluminum mirror was used as a 100% reflection reference, and the reflectance spectra were measured relative to the standard reference. The morphologies of hollow spheres were characterized by an FEI Tecnai G2 F30 transmission electron microscope (TEM). The microstructures of the photonic crystals were characterized by a Hitachi S4800 scanning electron microscope (SEM). The optical microscopy images were taken using an Olympus BXFM reflection-type microscope in dark-field mode. The finite element simulation was carried out using COMSOL Multiphysics.

## Data Availability

All data needed to evaluate the conclusions in the paper are present in the paper and/or the Supplementary Materials. Additional data related to this paper may be requested from the authors.

## Supplementary Materials

Supplementary Materials Figure S1: TEM image of (a) SiO_2_ colloidal particles and (b) SiO_2_@RF colloidal particles. Figure S2: larger scale TEM images of hollow RF spheres with diameters of (a) 210 nm, (b) 245 nm, and (c) 275 nm. Figure S3: the lightness coordinate of PC films during ethanol diffusion according to the Lab color space. Figure S4: the intensity signals during ethanol diffusion in PC films that were aged for 1 min (a), 5 min (b), 10 min (c), and 60 min (d) at 70°C. Figure S5: TEM images of (a–c) SiO_2_@RF with different shell thickness and (d–e) hollow RF spheres after etching the SiO_2_ core; (g–i) DRS patterns of ethanol diffusion process in PC films made from above hollow RF spheres. Figure S6: experimental and simulation time-dependent reflectance during the swelling step of DMSO in PCs. Figure S7: digital photos of PC films infiltrated by 1-pentanol, 1-hexanol, 1-heptanol, 1-octanol, 1,4-butanediol, and 1,3-butanediol. Table S1: the dielectric constant, viscosity, and refractive index of solvents. Table S2: the experimental and calculated wavelength shift caused by the swelling of different solvents. Only the change of RI was considered in the calculation. Video S1: diffusion of ethanol in hollow sphere photonic crystals recorded by a digital camera. Video S2: diffusion of ethanol in hollow sphere photonic crystals recorded by optical microscope. Video S3: diffusion of methanol in hollow sphere photonic crystals recorded by a digital camera.
