# Supplementary material for: High-Precision Colorimetric Sensing by Dynamic Tracking of Solvent Diffusion in Hollow-Sphere Photonic Crystals
Source: Research (Wash D C). 2022 May 6;2022:9813537. doi: 10.34133/2022/9813537 (PMC9107592; doi:10.34133/2022/9813537)
Supplement: Supplementary Materials — Figure S1: TEM image of (a) SiO2 colloidal particles and (b) SiO2@RF colloidal particles. Figure S2: larger scale TEM images of hollow RF spheres with diameters of (a) 210 nm, (b) 245 nm, and (c) 275 nm. Figure S3: the lightness coordinate of PC films during ethanol diffusion according to the Lab color space. Figure S4: the intensity signals during ethanol diffusion in PC films that were aged for 1 min (a), 5 min (b), 10 min (c), and 60 min (d) at 70°C. Figure S5: TEM images of (a–c) SiO2@RF with different shell thickness and (d–e) hollow RF spheres after etching the SiO2 core; (g–i) DRS patterns of ethanol diffusion process in PC films made from above hollow RF spheres. Figure S6: experimental and simulation time-dependent reflectance during the swelling step of DMSO in PCs. Figure S7: digital photos of PC films infiltrated by 1-pentanol, 1-hexanol, 1-heptanol, 1-octanol, 1,4-butanediol, and 1,3-butanediol. Table S1: the dielectric constant, viscosity, and refractive index of solvents. Table S2: the experimental and calculated wavelength shift caused by the swelling of different solvents. Only the change of RI was considered in the calculation. Video S1: diffusion of ethanol in hollow sphere photonic crystals recorded by a digital camera. Video S2: diffusion of ethanol in hollow sphere photonic crystals recorded by optical microscope. Video S3: diffusion of methanol in hollow sphere photonic crystals recorded by a digital camera. [file 9813537.f1.zip › Supplemental Material.docx]

**Supporting Information**

**High-Precision Colorimetric Sensing by Dynamic Tracking of Solvent Diffusion in Hollow-Sphere Photonic Crystals**

Qianqian Fu^1,2^, Jianping Ge^2^*, Chen Chen^1^, Zichen Wang^1^, Fan Yang^1^, Yadong Yin^1^*

1. Department of Chemistry, University of California, Riverside, California, USA, CA 92521

2. School of Chemistry and Molecular Engineering, Shanghai Key Laboratory of Green Chemistry and Chemical Processes, East China Normal University, Shanghai, China, 200062.


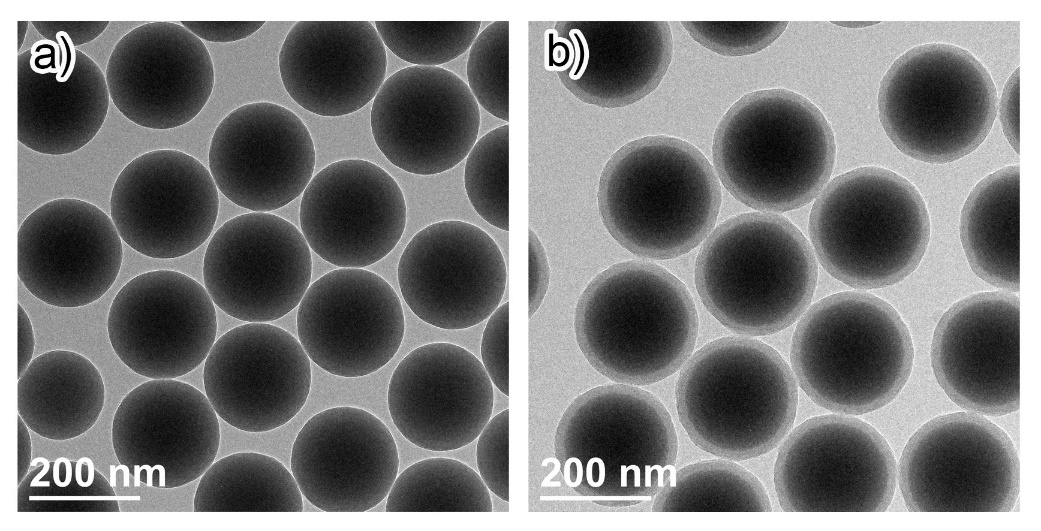


**Figure S1**. TEM image of a) SiO_2_ colloidal particles and b) SiO_2_@RF colloidal particles.


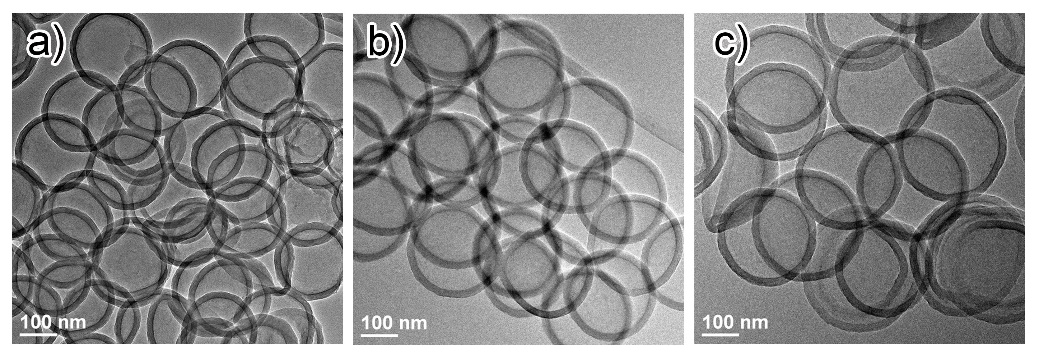


**Figure S2.** TEM images of hollow RF spheres with diameters of a) 210 nm, b) 245 nm, and c) 275 nm


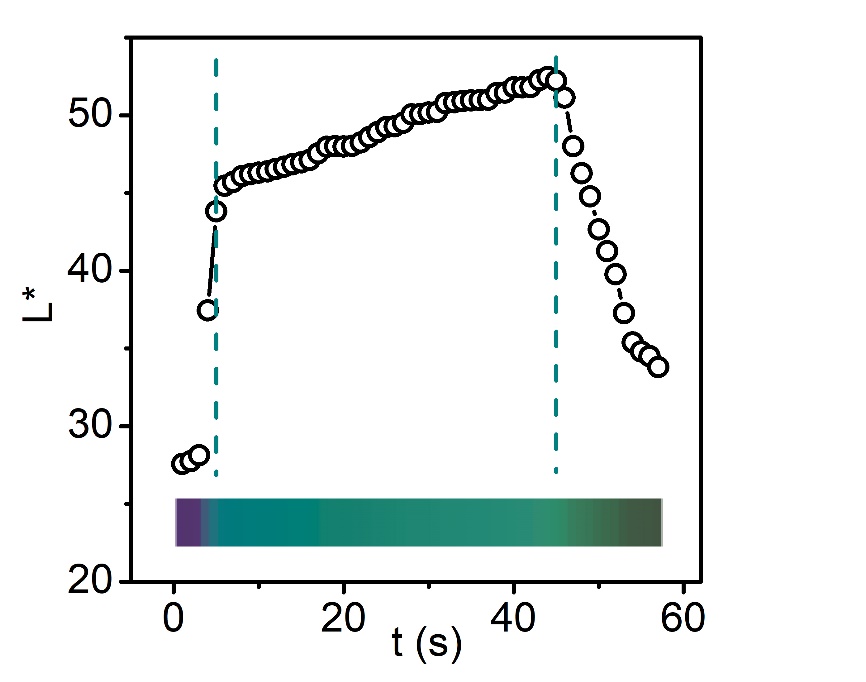


**Figure S3.** The lightness coordinate of PC films during ethanol diffusion according to the Lab color space, and the inset is the simulated color obtained by the color spectrophotometer.


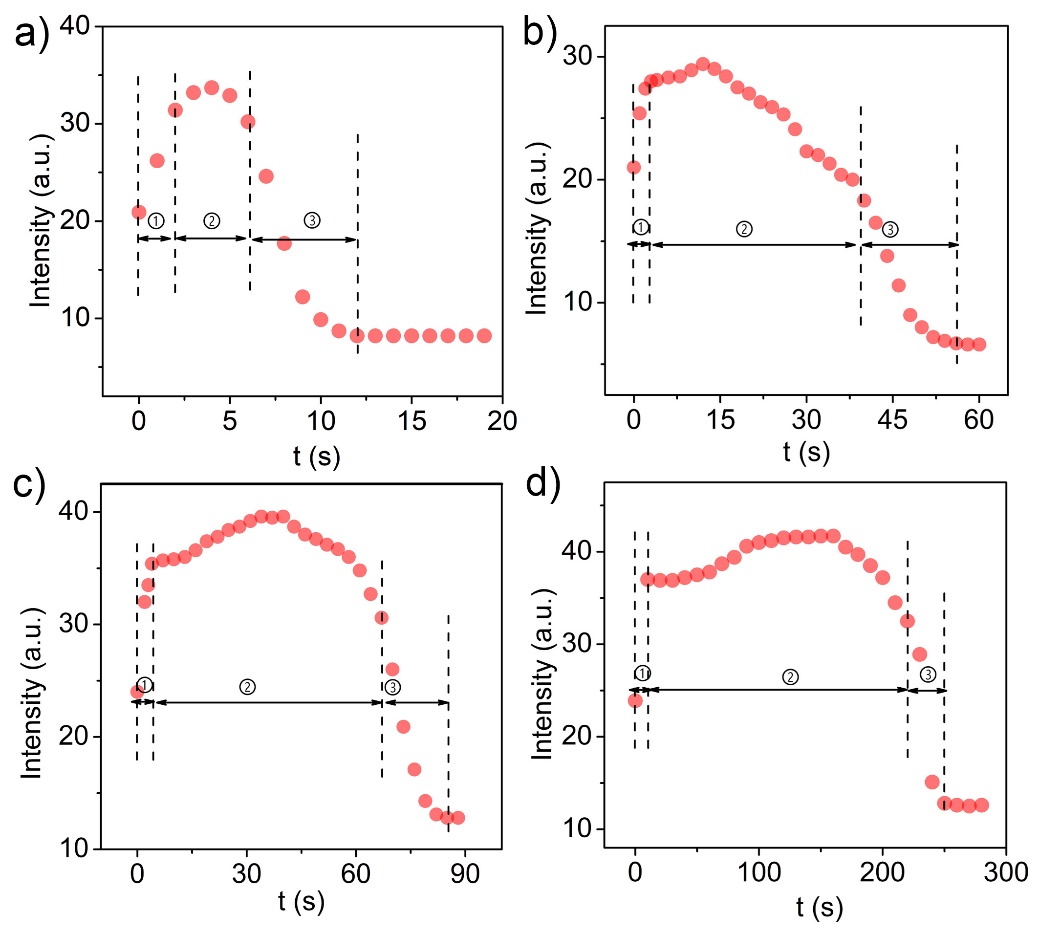


**Figure S4.** The intensity signals during ethanol diffusion in PC films that were aged for 1 min (a), 5 min (b), 10 min (c), and 60 min (d) at 70 °C.


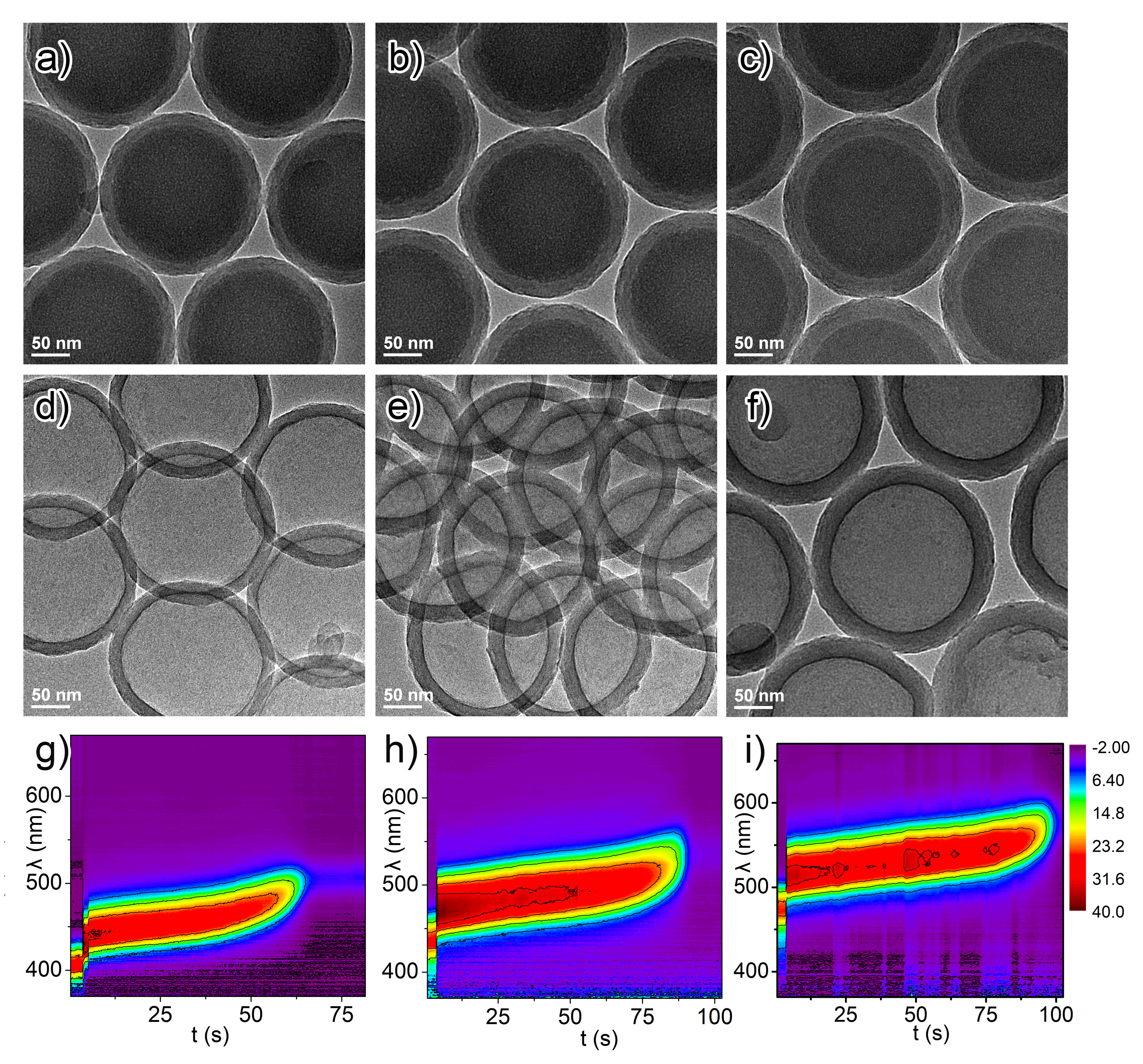


**Figure S5.** TEM images of a-c) SiO_2_@RF with different shell thickness and d-e) hollow RF spheres after etching the SiO_2_ core; g-i) DRS patterns of ethanol diffusion process in PC films made from above hollow RF spheres.


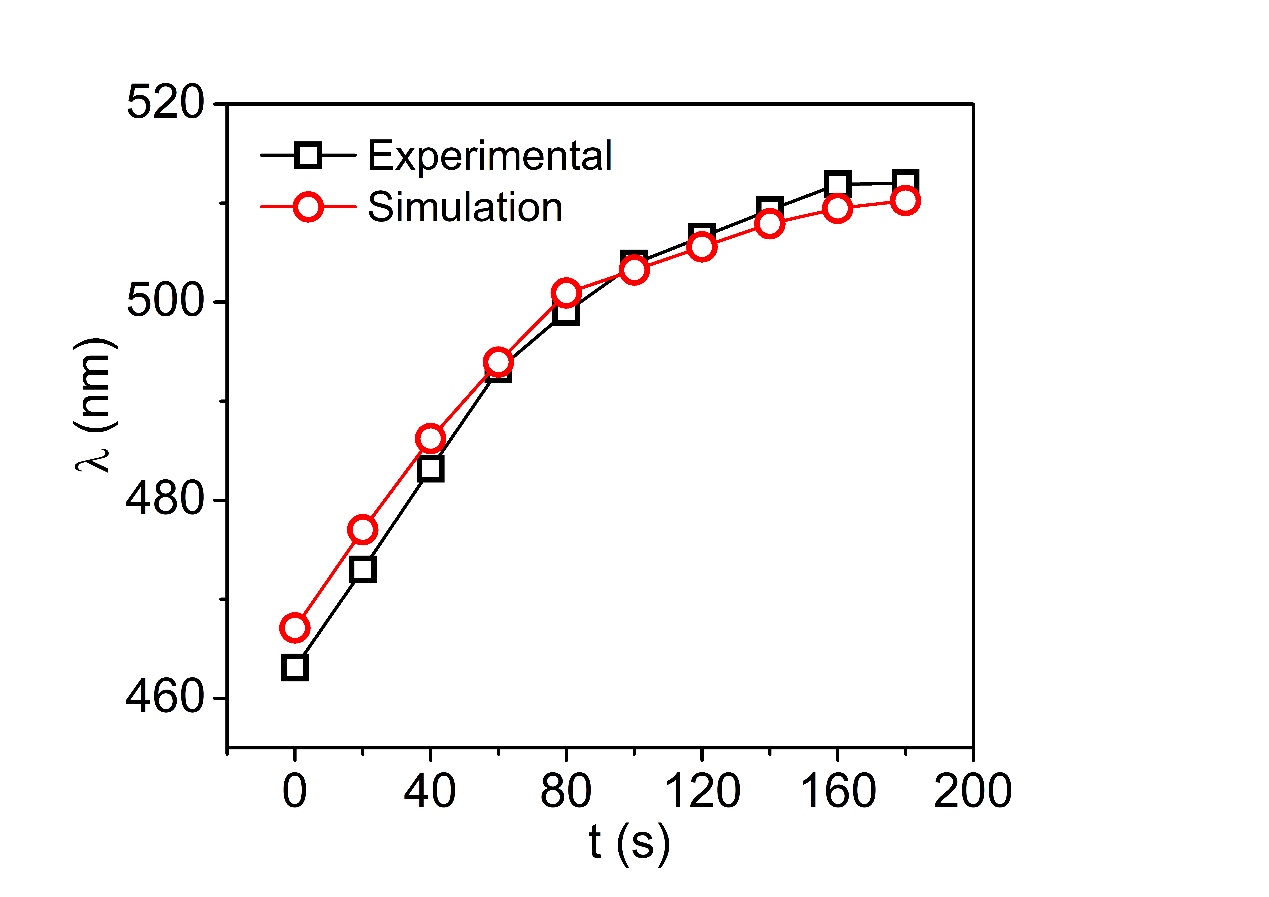


**Figure S6**. Experimental and simulation time-dependent reflectance during the swelling step of DMSO in PCs.


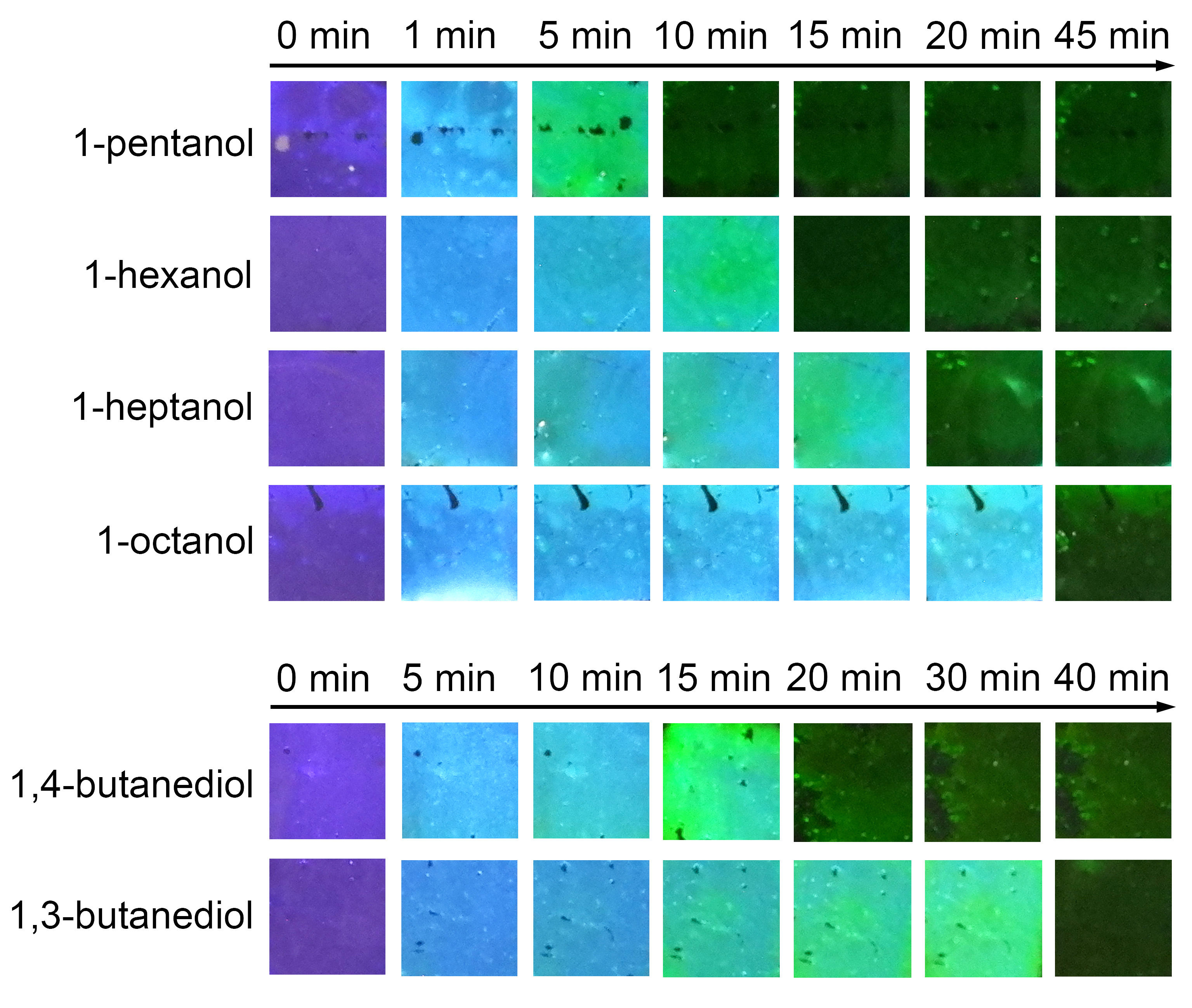


**Figure S7.** Digital photos of PC films infiltrated by 1-pentanol, 1-hexanol, 1-heptanol, 1-octanol, 1,4-butanediol and 1,3-butanediol. Here, the analytes were mixed with methanol in the proportion of 1:1 to speed up the measurement process.

**Table S1:** The dielectric constant, viscosity, and refractive index of solvents.

| Solvent | ε | Viscosity（mPa∙s） | RI |
| --- | --- | --- | --- |
| Methanol | 31.2 | 0.595 | 1.329 |
| Ethanol | 25.7 | 1.17 | 1.361 |
| 1-propanol | 20.3 | 2.26 | 1.386 |
| Iso-propanol | 19.92 | 2.4 | 1.377 |
| 1-butanol | 17.1 | 2.95 | 1.399 |
| 2-butanol | 15.5 | 4.210 | 1.397 |
| i-butanol | 17.95 | 4.000 | 1.396 |
| t-butanol | 11.4 | 3.35 | 1.384 |
| EG | 38.66 | 25.66 | 1.432 |
| DEG | 31.69 | 35.7 | 1.448 |
| Acetonitrile | 37.5 | 0.375 | 1.344 |
| DMSO | 48.9 | 1.996 | 1.477 |

**Table S2.** The experimental and calculated wavelength shift caused by the swelling of different solvents. Only the change of RI was considered in the calculation.

|  | Δλ_experiment_ | Δλ_Calculation_ |
| --- | --- | --- |
| H_2_O | 80.1 | 82.50 |
| Methanol | 93.4 | 81.51 |
| Ethanol | 97.8 | 89.44 |
| 1-propanol | 102.7 | 93.45 |
| Isopropanol | 103.5 | 95.63 |
| 1-butanol | 112.3 | 98.85 |
| EG | 129.1 | 107.03 |
| DEG | 132.3 | 110.99 |
| DMSO | 181.4 | 118.18 |
